# Supplementary material for: Assessing and assuring interoperability of a genomics file format
Source: Bioinformatics. 2022 May 16;38(13):3327–36. doi: 10.1093/bioinformatics/btac327 (PMC9237710; doi:10.1093/bioinformatics/btac327)
Supplement: btac327_Supplementary_Data [file btac327_supplementary_data.pdf]

Supplementary tables for "Assessing and assuring interoperability of a genomics file format"

Yi Nian Niu (0000-0002-5691-8238)[1], Eric G. Roberts (0000-0001-5398-358X)[1],
Danielle Denisko (0000-0002-8544-0026)[1,2], and
Michael M. Hoffman (0000-0002-4517-1562)[1,2,3,4,*]

[1]Princess Margaret Cancer Centre, University Health Network, Toronto, ON, Canada
[2]Department of Medical Biophysics, University of Toronto, Toronto, ON, Canada
[3]Department of Computer Science, University of Toronto, Toronto, ON, Canada
[4]Vector Institute, Toronto, ON, Canada

[*]Corresponding author

March 28, 2022

Tools passing	Test case
37	other-fully_space_delimited.bed
37	other-not_tab_delimited.bed
52	other-comment_start.bed
57	other-space_before_tab.bed
58	other-space_after_tab.bed
58	other-space_between_lines.bed
60	other-multiple_comments.bed
61	other-comment_end.bed
62	other-comment_middle.bed
67	03-start_eq_end.bed
71	01-known_scaffolds.bed
71	01-unknown_scaffolds.bed
78	02-leading_zeros_start.bed
79	03-duplicate_rows.bed
79	03-overlapping_intervals.bed
79	03-zero_chromStart.bed
79	03-no_gap_startend.bed
79	03-standard_chrom.bed

Supplementary Table 1: Number of tools that passed each of 18 expected success BED3 test cases. We tested 99 tools. The leading number in the test case name describes the Browser Extensible Data (BED) field that the test focuses on. "Other" means the test focuses on the structure of the file, rather than a particular field.

Test case name	Description
Field 1: chrom: the name of the chromosome where the feature is present.	
01-known_scaffolds	Scaffolds with a known chromosome such as “chr19_GL383573v1_alt”
01-unknown_scaffolds	Scaffolds with an unknown chromosome such as “chrUn_KI270442v1”
Field 2: chromStart: start position of the feature on the chromosome.	
02-leading_zeros_start	chromStart left-padded with “0”
Field 3: chromEnd: end position of the feature on the chromosome.	
03-duplicate_rows	Two features have the exact same (chrom, chromStart, chromEnd)
03-no_gap_startend	No gap between chromEnd of one feature and chromStart of the next feature
03-overlapping_intervals	All features’ intervals overlap each other
03-standard_chrom	A BED file without edge cases
03-start_eq_end	chromStart equals chromEnd
03-zero_chromStart	chromStart is “0”
Field 4: name: string that describes the feature.	
04-name_dot	All features have name “.”
04-repeated_name	Multiple features have the same non-dot name
04-special_character_name	Name contains non-alphanumeric ASCII characters
Field 5: score: integer between 0 and 1000, inclusive.	
05-edge_score	Score is “0” or “1000”
05-leading_zeros_score	Score left-padded with “0”
05-zero_scores	Score is “0”
Field 6: strand: strand that the feature appears on.	
06-dot_strand	Strand is “.”
06-mix_strand	Strand selected from one of “+”, “-”, or “.”
Field 7: thickStart: start position at which the feature is visualized with thicker display.	
07-leading_zeros_thickStart	thickStart left-padded with “0”
Field 8: thickEnd: end position at which the feature is visualized with thicker display.	
08-all_thick	thickStart equal to chromStart; thickEnd equal to chromEnd
08-no_thick_part	thickStart equals to thickEnd equal to chromStart
Field 9: itemRgb: A triplet of integers that determines the color of this feature when visualized.	
09-leading_zeros_colors	Color left-padded with “0”
09-many_colors	Multiple colors in the file
09-zero_rgb	Color is “0”

Supplementary Table 2: Expected success test cases.

Test case name	Description
Field 10: blockCount: number of blocks in the feature.	
`10-different_blockcounts`	Features have different numbers of blocks
`10-leading_zero_blockcount`	blockCount left-padded with “0”
`10-max_blocks`	Maximum number of blocks possible for its length
`10-one_block`	All features have only one block
Field 11: blockSizes: list of length blockCount containing the size of each block.	
`11-leading_zero_blocksize`	blockSizes list has a size left-padded with “0”
`11-length_one_block`	All blocks have a size of 1
`11-trailing_commas`	blockSizes list with a trailing comma
Field 12: blockStarts: list of length blockCount containing each block’s start position.	
`12-no_gap_blocks`	No gaps between blocks
Other: unrelated to individual BED fields.	
`other-comment_end`	Comment line at the end of the file
`other-comment_middle`	Comment line in the middle of the file
`other-comment_start`	Comment line at the start of the file
`other-fully_space_delimited`	Fields all space-delimited
`other-multiple_comments`	Many comments in various positions in the file
`other-not_tab_delimited`	File contains both space and tab delimiters
`other-space_after_tab`	Extra space after a tab delimiter
`other-space_before_tab`	Extra space before a tab delimiter
`other-space_between_lines`	Empty line between features

Supplementary Table 2: Expected success test cases. (Continued)

Test case name	Description
Field 1: chrom: the name of the chromosome where the feature is present.	
`01-missing_chrom`	chrom is “.”
`01-no_chrom`	Missing chrom
`01-special_char_chrom`	chrom has non-alphanumeric character
`01-quote_chrom`	chrom enclosed with quotation marks
Field 2: chromStart: start position of the feature on the chromosome.	
`02-float_start`	chromStart is floating-point number
`02-no_start`	Missing chromStart
`02-start_negative`	chromStart is negative
`02-string_start`	chromStart is not numeric

Supplementary Table 3: Expected fail test cases.

Test case name	Description
Field 3: chromEnd: end position of the feature on the chromosome.	
03-chromEnd_too_large	chromEnd exceeds maximum size of an unsigned integer
03-end_negative	chromEnd is negative
03-float_end	chromEnd is floating-point number
03-no_end	Missing chromEnd
03-start_gt_end	chromStart greater than chromEnd
03-string_end	chromEnd is not numeric
Field 4: name: string that describes the feature.	
04-mixed_bed_types	Features have a different number of fields
04-name_with_spaces	Name has a space
04-no_name	Missing name
Field 5: score: integer between 0 and 1000, inclusive.	
05-float_score	Score is floating-point number
05-no_score	Missing score
05-score_gt_1000	Score greater than 1000
05-score_lt_0	Score less than 0
05-string_score	Score is not numeric
Field 6: strand: strand that the feature appears on.	
06-invalid_strand	Strand not one of "+", "-", or "."
06-no_strand	Missing strand
Field 7: thickStart: start position at which the feature is visualized with thicker display.	
07-float_thickStart	thickStart is floating-point number
07-no_thickStart	Missing thickStart
07-string_thickStart	thickStart is not numeric
07-thickStart_lt_chromStart	thickStart less than chromStart
Field 8: thickEnd: end position at which the feature is visualized with thicker display.	
08-float_thickEnd	thickEnd is floating-point number
08-string_thickEnd	thickEnd is not numeric
08-thickEnd_gt_chromEnd	thickEnd greater than chromEnd
08-thickStart_gt_thickEnd	thickStart greater than thickEnd
08-thickStartEnd_wide	Thick interval not contained within [chromStart, chromEnd)
Field 9: itemRgb: A triplet of integers that determines the color of this feature when visualized.	
09-incomplete_rgb	RGB value not a triplet or "0"
09-no_rgb	Missing itemRgb
09-string_rgb	itemRgb contains alphabetical characters

Supplementary Table 3: Expected fail test cases. (Continued)

Test case name	Description
Field 10: blockCount: number of blocks in the feature.	
10-blockCount_negative	blockCount is negative
10-blockCount_string	blockCount is non-numeric
10-blockCount_zero	blockCount is "0"
10-no_blockCount	Missing blockCount
Field 11: blockSizes: list of length blockCount containing the size of each block.	
11-block_outofrange	Block located outside the interval chromStart to chromEnd
11-blocksizes_missing_comma	blockSizes list missing comma separators
11-mismatch_count_long	blockSizes list longer than blockCount
11-mismatch_count_short	blockSizes list shorter than blockCount
11-size_negative	Block has negative size
Field 12: blockStarts: list of length blockCount containing each block's start position.	
12-bed13	Feature has 13 fields
12-overlapping_blocks	Blocks overlap each other
12-start_outofrange	Block start position past chromEnd
12-unsorted_block_starts	blockStarts list not sorted
Other: unrelated to individual BED fields.	
other-quote_chrom	Feature enclosed in quotation marks

Supplementary Table 3: Expected fail test cases. (Continued)
